# Emergence in southern France of a new SARS-CoV-2 variant harbouring both N501Y and E484K substitutions in the spike protein

**DOI:** 10.1007/s00705-022-05385-y

**Published:** 2022-02-18

**Authors:** Philippe Colson, Jérémy Delerce, Emilie Burel, Jordan Dahan, Agnès Jouffret, Florence Fenollar, Nouara Yahi, Jacques Fantini, Bernard La Scola, Didier Raoult

**Affiliations:** 1 IHU Méditerranée Infection, 19-21 boulevard Jean Moulin, 13005 Marseille, France; 2 Institut de Recherche pour le Développement (IRD), Microbes Evolution Phylogeny and Infections (MEPHI), Aix-Marseille Université, 27 boulevard Jean Moulin, 13005 Marseille, France; 3 Assistance Publique-Hôpitaux de Marseille (AP-HM), 264 rue Saint-Pierre, 13005 Marseille, France; 4 Laboratoire de Biologie Médicale, Synlab Provence Marseille, 25 rue Rabattu, 13015 Marseille, France; 5 Laboratoire de Biologie Médicale Synlab Provence Forcalquier, rue du Souvenir Français, 04300 Forcalquier, France; 6 Institut de Recherche pour le Développement (IRD), Vecteurs-Infections Tropicales et Méditerranéennes (VITROME), Aix-Marseille Université, 27 boulevard Jean Moulin, 13005 Marseille, France; 7 Aix-Marseille Université, INSERM UMR S 1072, 51 boulevard Pierre Dramard, 13015 Marseille, France

## Abstract

SARS-CoV-2 variants have become a major virological, epidemiological, and clinical concern, particularly with regard to the risk of escape from vaccine-induced immunity. Here, we describe the emergence of a new variant, with the index case returning from travel in Cameroon. For 13 SARS-CoV-2-positive patients living in the same geographical area of southeastern France, a qPCR test for screening variant-associated mutations showed an atypical combination. The genome sequences were obtained by next-generation sequencing with Oxford Nanopore Technologies on GridION instruments within about 8 h. Analysis revealed 46 nucleotide substitutions and 37 deletions, resulting in 30 amino acid substitutions and 12 deletions. Fourteen of the amino acid substitutions, including N501Y and E484K, and nine deletions are located in the spike protein. This genotype pattern led to the establishment of a new Pangolin lineage, named B.1.640.2, that is a phylogenetic sister group to the old B.1.640 lineage, which has now been renamed B.1.640.1. The lineages differ by 25 nucleotide substitutions and 33 deletions. The combination of mutations in these isolates and their phylogenetic position indicate, based on our previous definition, that they represent a new variant, which we have named “IHU”. These data are a further example of the unpredictability of the emergence of SARS-CoV-2 variants, and of their possible introduction into a given geographical area from abroad.

**Supplementary Information:**

The online version contains supplementary material available at 10.1007/s00705-022-05385-y.

SARS-CoV-2 emerged in China in December 2019 and was declared a pandemic 21 months ago [1]. We have shown since the summer of 2020 that several SARS-CoV-2 variants have emerged in southeastern France and have caused distinct epidemics, either successive or superimposed [2, 3]. We also reported that these variants were often introduced from abroad but could also be mink. As of December 31, 2021, in our institute, SARS-CoV-2 from almost 43,000 patients had been genotyped, by next-generation sequencing (NGS) of the complete genomes for more than 23,000 patients, and by implementing multiple qPCR specific for each variant for a more exhaustive assessment of their spread. Since then, and with the emergence of the Alpha variant at the end of 2020, SARS-CoV-2 variants have become a major virological, epidemiological, and clinical concern, particularly regarding the risk of escape from vaccine-induced immunity [4–7]. Here, we describe the emergence in southeastern France of a new variant of possible Cameroonian origin.

The index case, aged between 40 and 50 and living in a small town in southeastern France, was first diagnosed as infected with SARS-CoV-2 by real-time reverse transcription PCR (qPCR) performed on a nasopharyngeal sample collected in mid-November 2021 at a private medical biology laboratory (Table 1). The person had been vaccinated against SARS-CoV-2 and had returned from travel to Cameroon three days previously. Mild respiratory symptoms arose the day before diagnosis. Subsequent detection of three mutations in the spike gene in a qPCR assay to screen for variants, as performed routinely in France in cases of SARS-CoV-2 positivity, revealed an atypical combination with L452R negativity, E484K positivity, and E484Q negativity (Pentaplex assay, ID solutions, Grabels, France), which did not correspond to the pattern of the Delta variant, which was associated with almost all SARS-CoV-2 infections at that time (Table 1). Respiratory samples collected from seven other SARS-CoV-2-positive patients living in the same geographical area of southern France exhibited the same combination of mutations in the qPCR assay used for screening. These patients included two adults and five children (<15 years of age) (Table 1). The respiratory samples from these eight patients were sent to the University Hospital Institute (IHU) Méditerranée Infection for SARS-CoV-2 genome sequencing as recommended by French public health authorities. A rapid NGS procedure was launched overnight, allowing SARS-CoV-2 genotype identification within about 8 hours. Briefly, viral RNA was extracted from 200 µL of nasopharyngeal swab fluid using a KingFisher Flex system (Thermo Fisher Scientific, Waltham, MA, USA), following the manufacturer’s instructions. Extracted RNA was reverse transcribed using SuperScript IV (Thermo Fisher Scientific), and a second cDNA strand was synthesized using a LunaScript RT SuperMix kit (New England Biolabs, Beverly, MA, USA) and then amplified using a multiplex PCR protocol according to the ARTIC procedure (https://artic.network/) with the ARTIC nCoV-2019 V3 panel of primers (IDT, Coralville, IA, USA). Finally, NGS was performed using a ligation sequencing kit and a GridION instrument from Oxford Nanopore Technologies (Oxford, UK), following the manufacturer’s instructions. Subsequently, fastq files were processed using the ARTIC field bioinformatics pipeline (https://github.com/artic-network/fieldbioinformatics). NGS reads were basecalled using Guppy (4.0.14) and aligned to the Wuhan-Hu-1 reference genome sequence (GenBank accession no. MN908947.3) using minimap2 (v2.17-r941) (https://github.com/lh3/minimap2) [8]. The ARTIC tool align_trim was used to softmask primers from the read alignment and to cap the sequencing depth at a maximum of 400. The identification of consensus-level variant candidates was performed using the Medaka (0.11.5) workflow (https://github.com/artic-network/artic-ncov2019). This strategy allowed assembly of the complete viral genome sequence from NGS reads obtained within 30 min of the run for cycle threshold (Ct) values of qPCR between 15 and 27. SARS-CoV-2 genomes were classified into Nextclade and Pangolin lineages using web applications (https://clades.nextstrain.org/; https://cov-lineages.org/pangolin.html) [9–11]. The sequences were deposited in the GISAID sequence database (https://www.gisaid.org/) [12] (Table 1). Phylogenies were reconstructed using the nextstrain/ncov tool (https://github.com/nextstrain/ncov) and visualized using Auspice (https://docs.nextstrain.org/projects/auspice/en/stable/). Respiratory samples collected before December 1, 2021, from five other SARS-CoV-2-positive patients living in the same city or borough as the index case could be identified by NGS as infected with the IHU variant (Table 1). The viral genome sequences from these patients were determined using the same procedure used for the eight first cases.

**Table 1:** Main epidemiological and virological features of cases identified with infection with the SARS-CoV-2 IHU variant

Case no.	Age	Epidemiological features	Date of respiratory sample collection	Diagnostic qPCR Ct	Results of qPCR used to screen for the presence of SARS-CoV-2 spike mutations	Results of the TaqPath COVID-19 qPCR assay (Targets: ORF1, S, and N genes)	Genome GISAID Id.
1	40^s^	Index case; travelled to Cameroon; family #1's case	16/11/2021	27	L452R-neg.; E484K-pos.; E484Q-neg.; N501Y-pos.; P681H-pos.	Pos. for all three genes	EPI_ISL_7156955
2	Child	Family #1's case	22/11/2021	21	L452R-neg.; E484K-pos.; E484Q-neg.; N501Y-pos.; P681H-pos.	Pos. for all three genes	EPI_ISL_7314302
3	Child	Family #2's case	22/11/2021	15	L452R-neg.; E484K-pos.; E484Q-neg.; N501Y-pos.; P681H-pos.	Pos. for all three genes	EPI_ISL_7381031
4	Child	Family #2's case	22/11/2021	18	L452R-neg.; E484K-pos.; E484Q-neg.; N501Y-pos.; P681H-pos.	Pos. for all three genes	EPI_ISL_7381062
5	40^s^	Family #2's case	24/11/2021	15	L452R-neg.; E484K-pos.; E484Q-neg.; N501Y-pos.; P681H-pos.	Pos. for all three genes	EPI_ISL_7156959
6	30^s^	Family #2's case	24/11/2021	17	L452R-neg.; E484K-pos.; E484Q-neg.; N501Y-pos.; P681H-pos.	Pos. for all three genes	EPI_ISL_7314417
7	Child	Family #2's case	27/11/2021	19	L452R-neg.; E484K-pos.; E484Q-neg.; N501Y-pos.; P681H-pos.	Pos. for all three genes	EPI_ISL_7314514
8	Child	-	26/11/2021	26	L452R-neg.; E484K-pos.; E484Q-neg.; N501Y-pos.; P681H-pos.	Pos. for all three genes	EPI_ISL_7314471
9	40^s^	-	25/11/2021	15	L452R-neg.; E484K-pos.; E484Q-neg.; N501Y-n.t..; P681H- n.t.	Pos. for all three genes	EPI_ISL_7552465
10	20^s^	-	25/11/2021	16	L452R-neg.; E484K-pos.; E484Q-neg.; N501Y-n.t..; P681H- n.t.	Pos. for all three genes	EPI_ISL_7552470
11	40^s^	-	01/12/2021	22	L452R-neg.; E484K-pos.; E484Q-neg.; N501Y-n.t..; P681H- n.t.	Pos. for all three genes	EPI_ISL_7552483
12	40^s^	-	30/11/2021	15	L452R-neg.; E484K-pos.; E484Q-neg.; N501Y-n.t..; P681H- n.t.	Pos. for all three genes	EPI_ISL_7601710
13	40^s^	-	01/12/2021	20	L452R-neg.; E484K-pos.; E484Q-neg.; N501Y-n.t..; P681H- n.t	Pos. for all three genes	EPI_ISL_7552486

Ct, cycle threshold value; Id., identifier; neg., negative; N, nucleocapsid; no., number; N.t., not tested; ORF1, open reading frame 1; pos., positive; qPCR, real-time reverse transcription PCR; S, spike. All 13 respiratory samples were collected between mid-November 2021 and early December 2021.

Analysis of the viral genome sequences revealed the presence of 46 nucleotide substitutions and 37 deletions, resulting in 30 amino acid substitutions and 12 deletions (Fig. 1a; Supplementary Tables S1 and S2). Fourteen amino acid substitutions and nine amino acid deletions were found in the spike protein. These include the substitutions N501Y and E484K, which are present in the Beta, Gamma, Theta, and Omicron variants [5, 13], F490S, which is present in the Lambda variant, and P681H, which is present in the Lambda and Omicron variants. In the other structural proteins, amino acid changes include two substitutions in the nucleocapsid protein and one in the membrane protein. In the non-structural proteins, the amino acid changes include one substitution each in the proteins Nsp2, Nsp4, Nsp6, Nsp12 (RNA-dependent RNA polymerase), and Nsp13 (helicase); two substitutions in Nsp14 (3’-5’exonuclease); and three deletions in Nsp6. Finally, in the regulatory proteins, amino acid changes include two substitutions in ORF3a, one in ORF8, and one in ORF9b. In addition, codon 27 of the ORF8 gene is changed to a stop codon, as in the Alpha variant [14]. Some members of the Marseille-4 variant lineage (B.1.160), which predominated in the Marseille geographical area between August 2020 and February 2021 [3], also exhibit a stop codon in the ORF8 gene, but at another position.

**Fig. 1:**
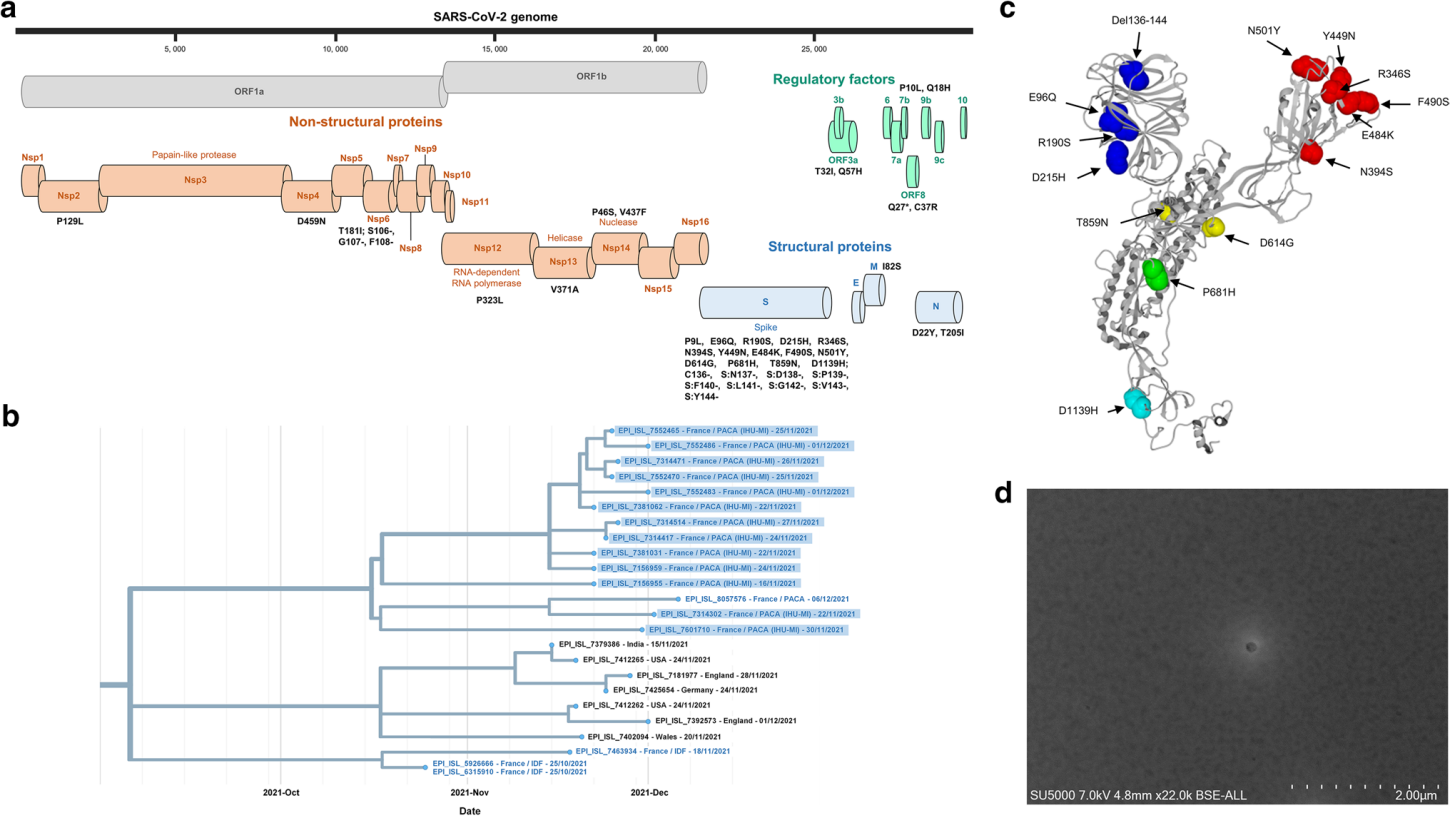
Virological features and scanning electron microscopy image of the SARS-CoV-2 IHU variant. (a) Map of the IHU variant genome showing amino acid substitutions and deletions. (b) Phylogeny reconstruction based on genome sequences of Pangolin lineage B.1.640.2 (available from the GISAID sequence database as of December 31, 2021). Phylogenetic analysis was performed using the nextstrain/ncov tool (https://github.com/nextstrain/ncov) and visualized using Auspice (https://docs.nextstrain.org/projects/auspice/en/stable/). The *x*-axis shows time. This figure is adapted from screenshots of the nextclade web application (https://clades.nextstrain.org) [9, 10]. Sequences are labelled with the GISAID identifier ((https://www.gisaid.org/) [12]), the country and region of origin, and the date of the patient’s sampling. ARA, Auvergne-Rhône-Alpes (French region); IDF, Ile-de-France (French region); IHU-MI, University Hospital Institute Méditerranée Infection (Marseille, France); PACA, Provence-Alpes-Côte d’Azur (French region). Sequences from France are shown in blue. Sequences obtained in our laboratory (IHU Méditerranée Infection, Marseille, France) are indicated by a pale blue background. (c) Representation of the spike of the IHU variant showing the location of all of its amino acid substitutions and deletions. N-terminal domain (NTD) mutations are in blue, receptor binding domain (RBD) mutations are in red, mutations involved in ACE-2 unmasking are in yellow, mutations at the S1-S2 cleavage site are in green, and mutations in the fusion region are in cyan. (d) Scanning electron microscopy image of a respiratory sample positive for the SARS-CoV-2 IHU variant, obtained using a SUV 5000 microscope (Hitachi High-Technologies Corporation, Tokyo, Japan)

Nextclade (https://clades.nextstrain.org/) identified a 20A lineage. Pangolin (https://cov-lineages.org/pangolin.html) identified a B.1.640 lineage in primary analysis but a B.1 lineage with the -usher (Ultrafast Sample placement on Existing tRee; https://genome.ucsc.edu/cgi-bin/hgPhyloPlace) option, which showed the phylogenetic placement of the genomes we obtained as an outgroup of the B.1.640 lineage and their clustering with a genome sequence obtained in late October in France (Ile-de-France) (EPI_ISL_5926666). The B.1.640 lineage corresponds to a variant first identified in France in April 2021, in Indonesia in August 2021, and in the Republic of the Congo (Brazzaville) in September 2021, and it was involved in a cluster of cases in Brittany, France, around mid-October 2021 [15]. As of December 31, 2021, 371 genome sequences were available from the GISAID database, including 275 from France and 29 from the Republic of the Congo. The sets of spike mutations in the B.1.640 lineage and in the genome sequences obtained here are similar, with 11 common nucleotide substitutions and one common deletion of nine codons (Supplementary Fig. S1, Supplementary Tables S1 and S2). However, the spike genes of these two lineages differ by seven mutations. In addition, 25 nucleotide substitutions and 33 nucleotide deletions located elsewhere in the genome differ between the two genotypes. The pattern of mutations therefore indicates that the sequences determined in this study represent a new variant, which we have named “IHU” (in reference to our institute), based on our previous definition [3]. A phylogenetic analysis performed using the nextstrain/ncov tool (https://github.com/nextstrain/ncov) also showed that the B.1.640 and IHU variants were most closely related to each other but comprised two divergent branches (Fig. 1b; Supplementary Fig. S2). Their last common ancestor was estimated to date from January 2021; however, there is no genome sequence currently available from GISAID that corresponds to it. Accordingly, a new Pangolin clade corresponding to the IHU variant was created on December 7, 2021, and named B.1.640.2, and the old B.1.640 clade was renamed B.1.640.1 (https://github.com/cov-lineages/pango-designation/issues/362). This clade encompassed (as of December 31, 2021) the present genomes and eleven others (Fig. 1b). Phylogeny reconstruction showed three major clusters. The first one included the 13 genomes obtained in our laboratory and one additional genome obtained in France in December 2021. A second cluster included seven genomes obtained from patients sampled in India, the United Kingdom, Germany, and the USA between mid-November and early December 2021. A third cluster included three genomes obtained from patients sampled in France in late October and mid-November 2021. As the index case was possibly infected with the IHU variant during his stay in Cameroon, we searched for this variant in GISAID among the genome sequences from this country, but as of December 31, 2021 none of the 556 available genome sequences belonged to the B.1.640.1 or B.1.640.2 lineage.

We analyzed a structural model of the complete spike protein of the IHU variant, generated by incorporating its specific mutational profile into the spike protein structure of the original 20B SARS-CoV-2 (Wuhan-Hu-1 isolate with the D614G substitution) [16] and fixing all gaps in the pdb file by incorporating the missing amino acids using the Robetta protein structure prediction tool [https://robetta.bakerlab.org/], followed by energy minimization using the Polak-Ribière algorithm as described previously (Fig. 1c) [17]. In the N-terminal domain (NTD), the deletion of amino acids 134-145 is predicted to significantly affect the neutralizing epitope. Other changes involve amino acids at positions 96 and 190: in the Wuhan-Hu-1 isolate, E96 and R190 induce a turn in the NTD secondary structure through electrostatic interactions with each other. This interaction is conserved between the substituted amino acids 96Q and 190S, which suggests the co-evolution of these changes. In the receptor binding domain (RBD), in addition to the well-known substitutions N501Y and E484K, several changes were predicted to significantly affect the neutralizing epitopes. In particular, P681H is located in the cleavage site of the S1-S2 subunits of the spike and is observed in other variants, including the recently emerging Omicron variant [13]. In addition, the D1139H substitution involves an amino acid that is involved in the fusion of the virus and infected cell. Also, D614G is combined with T859N in the IHU variant. Interestingly, in the Wuhan-Hu-1 isolate, the amino acids D614 and T859 from two subunits of the trimeric spike are face to face and lock the trimer in a closed conformation. Although the D614G substitution already allows the trimer conformation to be unlocked, this is predicted to be facilitated even more in the presence of the additional substitution T859N.

Seven patients were involved in intrafamilial cases, two being the index case and a relative whose viral genome exhibited seven nucleotide differences (99.98% identity). All 13 IHU-variant-positive samples showed the same combination of spike mutations identified using real-time qPCR techniques: negativity for 452R and 484Q, positivity for 484K, and, when tested, positivity for 501Y [18] and 681H. We also used a TaqPath COVID-19 kit (Thermo Fisher Scientific, Waltham, USA), which gave positive signals for all three genes targeted (ORF1, S, and N). Thus, the IHU variant could be distinguished in qPCR screening assays from the Delta variant (L452R positive) and the Omicron variant (L452R negative and negative for S gene detection by the TaqPath COVID-19 assay) co-circulating in southern France. Finally, scanning electron microscopy using an SUV 5000 microscope (Hitachi High-Technologies Corporation, Tokyo, Japan) [19] allowed a quick visualization of the virus from a respiratory sample (Fig. 1d).

Overall, these observations show once again the unpredictability of the emergence of new SARS-CoV-2 variants and their possible introduction from abroad, and they exemplify the difficulty in controlling such introductions and subsequent spread. They also confirm the value of the SARS-CoV-2 genomic surveillance that we started at the very beginning of the pandemic in the Marseille geographical area as soon as we diagnosed the first SARS-CoV-2 infection [19] and that we expanded during the summer of 2020 [2, 3]. Such surveillance program was implemented at the national level in 2021 through the French Emergen consortium (https://www.santepubliquefrance.fr/dossiers/coronavirus-covid-19/consortium-emergen). It is too early to speculate on the virological, epidemiological, or clinical features of this IHU variant based on these 13 cases. For this purpose, respiratory samples from infected patients were inoculated onto Vero E6 cells as described previously [20] in order to assess the susceptibility of this variant to neutralization by anti-spike antibodies elicited by vaccination or prior infection [21].

## Supplementary Information

Below is the link to the electronic supplementary material.

Supplementary file1 (DOCX 362 KB)

Supplementary file2 (DOCX 362 KB)

## Data Availability

The dataset generated and analyzed during the current study is available in the GISAID database (https://www.gisaid.org/).
