# Supplementary material for: Emergence in southern France of a new SARS-CoV-2 variant harbouring both N501Y and E484K substitutions in the spike protein
Source: Arch Virol. 2022 Feb 18;167(4):1185–90. doi: 10.1007/s00705-022-05385-y (PMC8853869; doi:10.1007/s00705-022-05385-y)
Supplement: Supplementary file 1 — Supplementary file1 (DOCX 362 KB) [file 705_2022_5385_MOESM1_ESM.docx]

**data deposition information**

**Name of the used repository:**

GISAID (Global Initiative on Sharing Avian Influenza Data) sequence database.

**Web link to the datasets:**

https://www.gisaid.org/

**Reference of the repository :**

Alm E, Broberg EK, Connor T, Hodcroft EB, Komissarov AB, Maurer-Stroh S, Melidou A, Neher RA, O'Toole A, Pereyaslov D, WHO European Region sequencing laboratories and GISAID EpiCoV group; WHO European Region sequencing laboratories and GISAID EpiCoV group (2020) Geographical and temporal distribution of SARS-CoV-2 clades in the WHO European Region, January to June 2020. Euro. Surveill 25: 2001410.

**Genome sequence identifiers in the GISAID database (repository): EPI_ISL_7156955**

EPI_ISL_7314302

EPI_ISL_7381031

EPI_ISL_7381062

EPI_ISL_7156959

EPI_ISL_7314417

EPI_ISL_7314514

EPI_ISL_7314471

EPI_ISL_7552465

EPI_ISL_7552470

EPI_ISL_7552483

EPI_ISL_7601710
