# Supplementary material for: Emergence in southern France of a new SARS-CoV-2 variant harbouring both N501Y and E484K substitutions in the spike protein
Source: Arch Virol. 2022 Feb 18;167(4):1185–90. doi: 10.1007/s00705-022-05385-y (PMC8853869; doi:10.1007/s00705-022-05385-y)
Supplement: Supplementary file 2 — Supplementary file2 (DOCX 362 KB) [file 705_2022_5385_MOESM2_ESM.docx]

**SUPPLEMENTARY MATERIAL**

**SUPPLEMENTARY FIGURE LEGENDS**

**Supplementary Figure S1. Microarray showing the distribution along the SARS-CoV-2 genome and in viral genes of nucleotide changes observed in comparison with the genome of the Wuhan-Hu-1 isolate for the Pangolin B.1.640.1 and B.1.640.2 (IHU variant) lineages.**


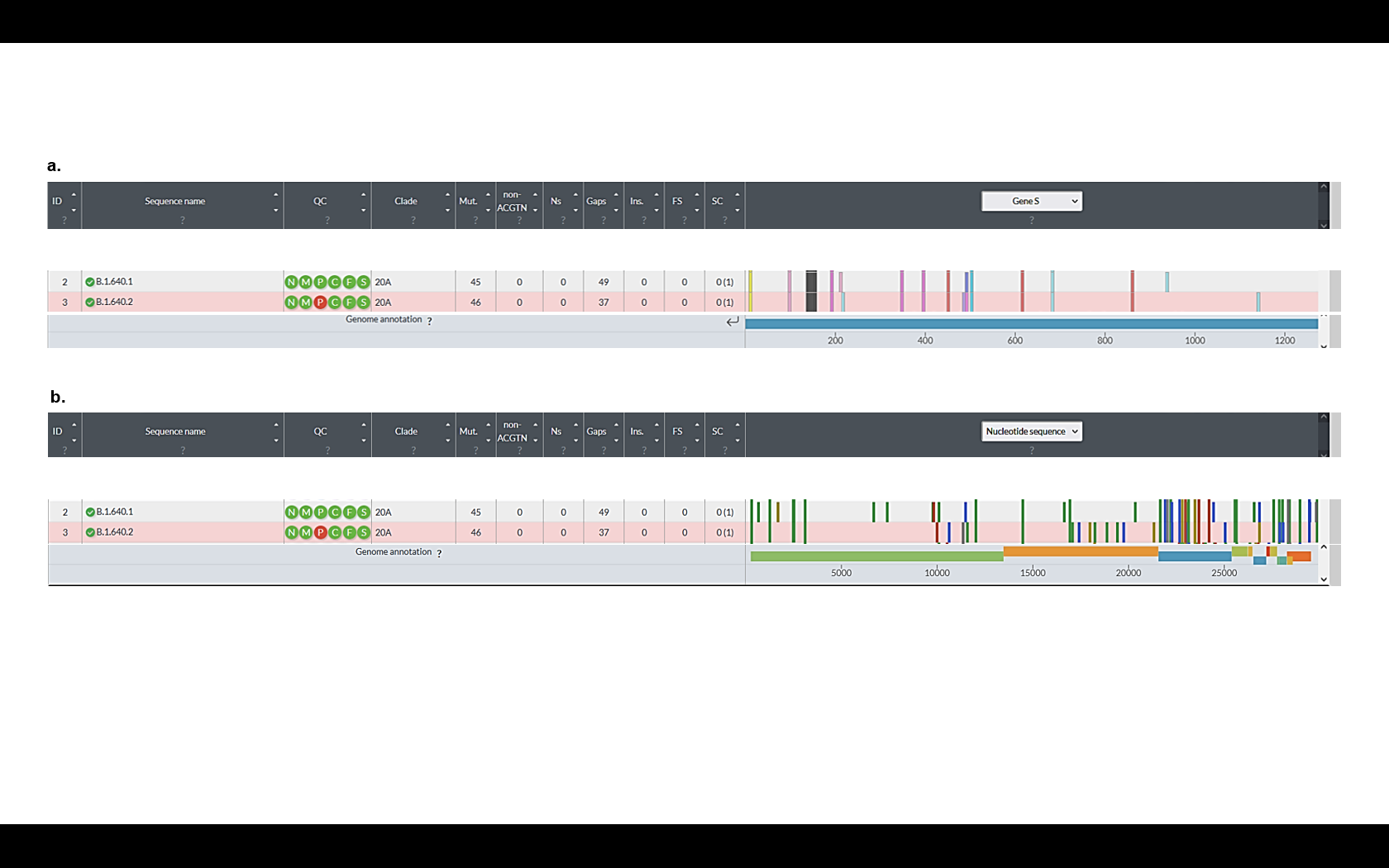


Genomes were analyzed using the Nextstrain web-tool (https://clades.nextstrain.org/) [1,2]. Representation is adapted from Nextclade sequence analysis web application output (https://clades.nextstrain.org/).

**Supplementary Figure S2. Phylogeny reconstruction based on genomes of Pangolin lineages B.1.640.1 and B.1.640.2.**

**Phylogenetic analysis was performed using the nextstrain/ncov tool (https://github.com/nextstrain/ncov) then visualized with Auspice (https://docs.nextstrain.org/projects/auspice/en/stable/). The genome of the original Wuhan-Hu-1 SARS-CoV-2 isolate (GenBank accession no. NC_045512.2) was added as outgroup, in addition to SARS-CoV-2 genomes of Pangolin lineage B.1.640.2 (available from the GISAID sequence database as of 31/12/2021) and to some SARS-CoV-2 genomes of Pangolin lineage B.1.640.1. X-axis shows time. This Figure is a screenshot of the nextclade web application (https://clades.nextstrain.org) [1, 2].**

**SUPPLEMENTARY TABLES**

**Supplementary Table S1. Comparison of nucleotide mutational patterns of the B.1.640.1 and B.1.640.2 lineages**

| B.1.640.1 | B.1.640.2 (IHU variant) |
| --- | --- |
| C241T | C241T |
| C601T | - |
| C1191T | C1191T |
| A1620G | - |
| C2416T | C2416T |
| C2455T | C2455T |
| C3037T | C3037T |
| G6622T | - |
| G7328T | - |
| C9711T | - |
| G9756A | - |
| - | G9929A |
| C10029T | - |
| - | T10561C |
| - | Deletion 11288-11296 |
| T11418C | - |
| - | C11514T |
| C11956T | C11956T |
| C14408T | C14408T |
| C16575T | - |
| C16869T | C16869T |
| - | C17004T |
| - | T17348C |
| - | A17916G |
| - | C18175T |
| - | C18804T |
| - | G19348T |
| - | T19680C |
| C20283T | - |
| - | A21258G |
| C21588T | C21588T |
| G21848C | G21848C |
| Deletion 21968-21994 | Deletion 21968-21994 |
| G22132T | G22132T |
| T22191C | - |
| - | G22205C |
| A22600C | A22600C |
| A22743G | A22743G |
| T22907A | T22907A |
| - | G23012A |
| T23030C | - |
| T23031**G** | T23031**C** |
| A23063T | A23063T |
| A23403G | A23403G |
| C23604A | C23604A |
| C24138A | C24138A |
| G24368C | - |
| - | G24977C |
| C25487T | C25487T |
| G25563T | G25563T |
| A26492T | - |
| T26767C | T26767G |
| C27513T | C27513T |
| C27807T | C27807T |
| - | T27833C |
| C27972T | C27972T |
| - | T28002C |
| Deletion 28271 | Deletion 28271 |
| T28297C | C28312T |
| G28337T | G28337T |
| C28887T | C28887T |
| T29377C | T29377C |
| G29405C | - |
| Deletion 29738-29758 | - |
| G29779T | G29779T |

Genomes were analyzed using the Nextstrain web-tool (https://clades.nextstrain.org/) [1,2].

Spike region is indicated by a grey background.

**Supplementary Table S2. Comparison of amino acid mutational patterns of the B.1.640.1 and B.1.640.2 lineages**

| B.1.640.1 | B.1.640.2 (IHU variant) |
| --- | --- |
| M:I82T | M:I82S |
| N:D22Y | N:D22Y |
| N:T205I | N:T205I |
| N:E378Q | - |
| ORF1a:P309L | ORF1a:P309L |
| ORF1a:E452G | - |
| ORF1a:L2119F | - |
| ORF1a:A2355S | - |
| ORF1a:S3149F | - |
| ORF1a:R3164H | - |
| - | ORF1a:D3222N |
| ORF1a:T3255I | - |
| - | Deletions ORF1a:S3675-, ORF1a:G3676-, ORF1a:F3677- |
| ORF1a:V3718A | - |
| - | ORF1a:T3750I |
| ORF1b:P314L | ORF1b:P314L |
| - | ORF1b:V1294A |
| - | ORF1b:P1570S |
| - | ORF1b:V1961F |
| ORF3a:T32I | ORF3a:T32I |
| ORF3a:Q57H | ORF3a:Q57H |
| ORF8:Q27* | ORF8:Q27* |
| ORF9b:I5T | - |
| ORF9b:Q18H | - |
| - | ORF8:C37R |
| - | ORF9b:P10L |
| - | ORF9b:Q18H |
| S:P9L | S:P9L |
| S:E96Q | S:E96Q |
| Deletions S:C136-, S:N137-, S:D138-, S:P139-, S:F140-, S:L141-, S:G142-, S:V143-, S:Y144- | Deletions S:C136-, S:N137-, S:D138-, S:P139-, S:F140-, S:L141-, S:G142-, S:V143-, S:Y144- |
| S:R190S | S:R190S |
| S:I210T | - |
| - | S:D215H |
| S:R346S | S:R346S |
| S:N394S | S:N394S |
| S:Y449N | S:Y449N |
| - | S:E484K |
| S:F490R | S:F490S |
| S:N501Y | S:N501Y |
| S:D614G | S:D614G |
| S:P681H | S:P681H |
| S:T859N | S:T859N |
| S:D936H | - |
| - | S:D1139H |

Genomes were analyzed using the Nextstrain web-tool (https://clades.nextstrain.org/) [1,2].

Spike region is indicated by a grey background.

**REFERENCES**

1. Hadfield J, Megill C, Bell SM, Huddleston J, Potter B, Callender C, Sagulenko P, Bedford T, Neher RA (2018). Nextstrain: real-time tracking of pathogen evolution. Bioinformatics 34: 4121-4123.

2. Aksamentov I, Roemer C, Hodcroft EB, Neher RA (2021). Nextclade: clade assignment, mutation calling and quality control for viral genomes. Zenodo https://doi.org/10.5281/zenodo.5607694.
